# Safety Evaluation of Oral Sirolimus in the Treatment of Childhood Diseases: A Systematic Review

**DOI:** 10.3390/children9091295

**Published:** 2022-08-26

**Authors:** Zixin Zhang, Yanan Li, Guangyue Zhang, Kaiying Yang, Tong Qiu, Jiangyuan Zhou, Xue Gong, Yi Ji

**Affiliations:** 1 Division of Oncology, Department of Pediatric Surgery, West China Hospital of Sichuan University, Chengdu 610041, China; 2 Med-X Center for Informatics, Sichuan University, Chengdu 610041, China

**Keywords:** sirolimus, safety, childhood diseases

## Abstract

**Background:** Sirolimus, a mammalian target of rapamycin inhibitor, has been widely used in pediatric patients, but the safety of sirolimus in pediatric patients has not been well determined. **Objective:** The objective of this study was to systematically evaluate prospective studies reporting the safety of sirolimus in the treatment of childhood diseases. **Methods:** The following data were extracted in a standardized manner: study design, demographic characteristics, intervention, and safety outcomes. **Results:** In total, 9 studies were included, encompassing 575 patients who received oral sirolimus for at least 6 months. Various adverse events occurred. The most common adverse event was oral mucositis (8.2%, 95% CI: 0.054 to 0.110). Through comparative analysis of the subgroups based on the targeted concentration range, we discovered that many adverse events were significantly higher in the high concentration group (≥10 ng/mL) than in the low concentration group (<10 ng/mL) (*p* < 0.01). More interestingly, we found that oral mucositis was more frequently reported in children with vascular anomalies than tuberous sclerosis complex. **Conclusions:** This study shows that oral sirolimus in the treatment of childhood diseases is safe and reliable. However, sirolimus treatment in the pediatric population should be strictly monitored to reduce the occurrence of serious or fatal adverse events.

## 1. Introduction

Sirolimus was first isolated by the fermentation of *Stretomyces hygroscopicus* from a soil sample in 1975 [1,2]. Although it was initially considered to be an antifungal agent [3], it was first approved as an anti-rejection medication in renal transplantation in the USA in 1999 [4]. Sirolimus has been found to inhibit mammalian target of rapamycin (mTOR) and bind to FK506-binding protein 12 (FKBP12) [5], which blocks cellular proliferation, especially in cell cycle progression from G1 phase to S phase [6,7]. From the time of its discovery, scholars have paid more attention to adults [3,8,9], and only a few studies have limited the factor of age and carried out studies on children. However, sirolimus is as widely used in children as in adults. The safety of sirolimus in vascular abnormalities was investigated in one previous study [8]; however, this study merely used a descriptive analysis. The safety of sirolimus in children has not been systematically investigated. As a result, the performance of a study in this field is vital.

mTOR, a serine/threonine kinase, is regulated by phosphoinositide 3 kinase (PI3K) and protein kinase B (Akt). mTOR plays an important role in numerous cellular processes, such as protein synthesis [9], angiogenesis [10], lipid biosynthesis [11], mitochondrial function [12], cell growth, and autophagy [13]. Therefore, the PI3K/AKT/mTOR pathway is essential for cellular metabolism and cell proliferation [8,14], both of which bring a new era of targeted molecular therapy for the use of sirolimus. Apart from transplantation, sirolimus shows beneficial effects in many other diseases, including rare diseases that seriously affect the quality of life of infants. For example, kaposiform hemangioendothelioma (a rare vascular neoplasm with high morbidity and mortality) [15,16,17,18], Olmsted syndrome (a rare and disabling genodermatosis) [19], and congenital hyperinsulinism (the most frequent cause of persistent hypoglycemia in infants) have been successfully treated with sirolimus. The patients tolerated sirolimus well and had obvious improvements in symptoms, quality of life, and/or shrinkage of the lesion. As long as a disease is found to be associated with the PI3K/AKT/mTOR pathway, sirolimus seems to have a certain curative influence.

The US Food and Drug Administration only approved sirolimus in children ≥ 13 years old; additionally, it was approved as an immunosuppressive agent after renal transplant [20]. This drug is commonly used off-label for specific ages and for indications in various childhood diseases [21]. However, the safety of sirolimus in pediatric patients has not been well evaluated. Therefore, we systematically evaluated prospective studies reporting the safety of sirolimus in the treatment of childhood diseases.

## 2. Method

The systematic review protocol was registered in PROSPERO (CRD42020213531) at https://www.crd.york.ac.uk/PROSPERO/, (accessed on 20 June 2022). This protocol was designed strictly according to the Preferred Reporting Items for Systematic reviews and Meta-Analyses (PRISMA) guidelines.

### 2.1. Search Strategy

The PubMed, MEDLINE, Embase, Cochrane Library, and Web of Science databases were searched up to 20 October 2021. The search terms contained Medical Subject Headings (MeSH) and free text words, including (“sirolimus” OR “rapamycin” OR “I-2190A” OR “I 2190A” OR “I2190A” OR “AY 22-989” OR “AY 22 989” OR “AY 22989” OR “rapamune”) AND (“child” OR “children” OR “pediatric patients” OR “infants”).

### 2.2. Inclusion Criteria and Exclusion Criteria

Studies were included if the following conditions were met: (1) the patients were aged ≤18 or the study was clearly identified as a pediatric study; (2) treatment with oral sirolimus; (3) studies with a prospective design; and (4) studies that had a clear description of adverse events (AEs). We excluded studies if they were (1) studies of topical therapies; (2) animal experiments; (3) duplicate publications; (4) publications with no detailed original data; (5) not publications; or (6) studies not in the English language.

### 2.3. Data Extraction

Two reviewers (ZXZ and YNL) independently searched the databases and extracted the following data using a data collection form discussed by both parties: (1) general information, including first author, publication year, journal, study design, diagnosis, sample size, number of males and females, age range, and median age; (2) treatment data, including starting dose, targeted blood concentrations, and treatment duration; and (3) AE data, including the name of the AEs and the occurrence number. If there was a difference in the data extraction process, a third researcher (YJ) was consulted to resolve discrepancies.

### 2.4. Differences between the Protocol and the Work That Was Performed

In the protocol, the age of the patients was limited to under 14 years of age because a prospective study with a large sample size defined the age as 0–14 years [22]. However, during the literature search, it was found that the age boundary of many prospective studies was 17 or 18 years old [23,24,25]. Therefore, the first change in the protocol was to change the age to 18 years old. Secondly, in the protocol, the types of studies were limited to randomized controlled trials and quasi-randomized trials. However, only 2 studies could be included when the types of studies were limited, and the other search terms remained the same as above. Therefore, to expand the range, the final search strategy did not limit the types of studies, and this constituted the second change in the protocol. Thirdly, gray literature databases were not searched because they did not include publications. This constituted the third change.

### 2.5. Quality Assessment

The risk of bias of RCTs was assessed using the Cochrane risk of bias tool of 2011 [26]. This tool comprised the following items: random sequence generation, allocation concealment, blinding of participants, personnel and outcome assessments, incomplete outcome data, selective reporting, and other bias. However, single-arm trials, including single-arm studies and experimental study arms, were assessed using the Newcastle Ottawa Scale (NOS) [27,28]. The NOS evaluates three aspects: selection, comparability, and outcome. Quality was categorized into three levels: poor (score, 0–3), fair (score, 4–6), or good (score, 7∼9).

### 2.6. Data Synthesis and Analysis

For each adverse reaction reported in more than two studies, a forest map and funnel map were constructed to obtain 95% confidence intervals and highly heterogeneous data. Studies with high heterogeneity refer to those studies that fall outside of the funnel triangle or those studies with black spots that are far away from the vertical line in the forest map. Heterogeneity across the included studies was examined by I^2^ statistics (significant heterogeneity, I^2^ > 50%; insignificant heterogeneity, I^2^ ≤ 50%). When I^2^ >50%, we used the random effects model and removed the study with highly heterogeneous data to make I^2^ ≤ 50% or close to 50%; subsequently, the fixed effects model was chosen. If necessary, sensitivity analyses were used to determine the reasons for heterogeneity. In addition, if some conditions, such as the targeted blood concentrations and diagnosis, significantly differed, we performed a subgroup analysis to determine whether this condition could affect the incidence of AEs. The cutoff for statistical significance was *p* < 0.05. All data were analyzed by R software (R version 4.1.0 ).

## 3. Results

### 3.1. Search Results and Characteristics of the Included Studies

**Figure 1:**
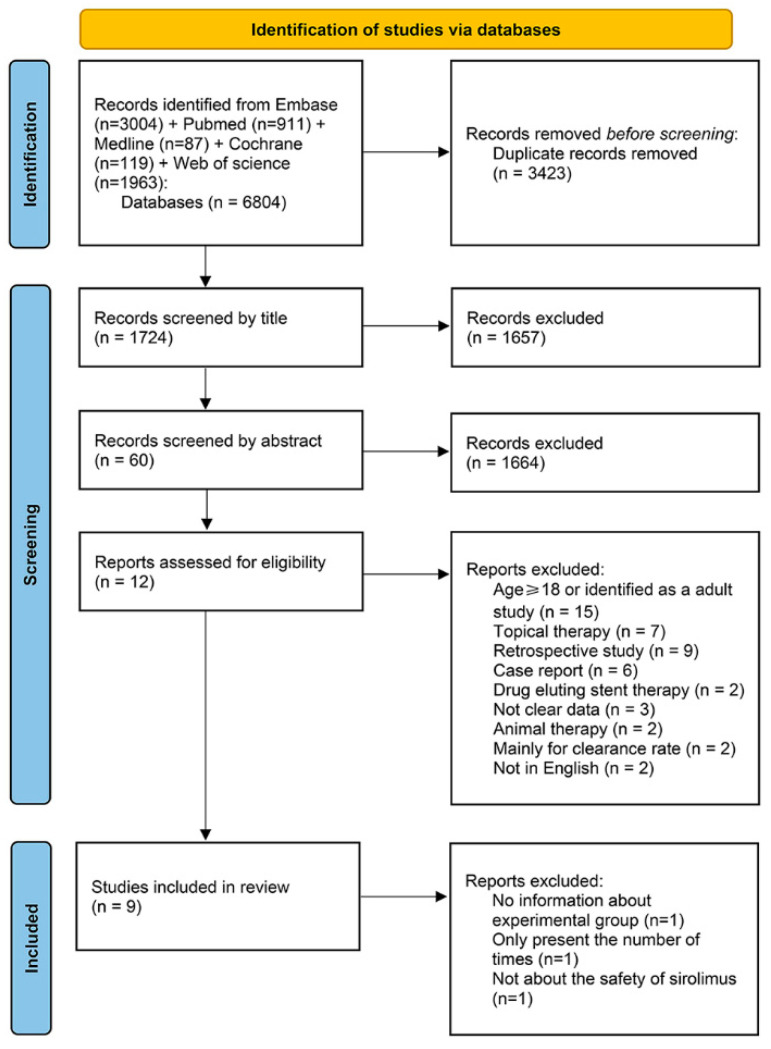
Flow chart of the literature search and study selection. Adapted from Page MJ, McKenzie JE, Bossuyt PM, et al. The PRISMA 2020 statement: an updated guideline for reporting systematic reviews. *Bmj.* 2021; 372: n71.

The detailed retrieval process for this protocol is revealed in Figure 1. In total, 6804 studies were identified after the initial keyword search. Through duplication, 3381 articles remained. Moreover, 1724 studies remained after scanning the titles and 60 studies remained after scanning the abstracts. After a careful full-text review, 12 studies were eligible for the final data extraction and analysis. Finally, nine studies [22,23,24,25,29,30,31,32,33] were included in the analyses. The remaining three studies were excluded, including a prospective cohort study and two randomized controlled trials. The prospective cohort study [34] only presented a table of the number of incidences rather than the number of cases. Additionally, for the two randomized controlled trials, one study [35] had no information about the experimental group, and this study only showed the AE data of the experimental and placebo groups together. The other study [36] mainly discussed patient tolerance of four sirolimus doses (Table S1).

The characteristics of the subsumed studies are summarized in Table 1. In total, the 9 studies consisted of 575 patients, and there were 7 prospective studies [22,23,24,29,30,31,32] and 2 randomized controlled trials [25,33]. Of these studies, four were related to tuberous sclerosis complex [25,31,32,35], two were related to lymphatic anomalies [31,32], two were related to vascular anomalies [22,25] and one was related to nephrotic syndrome [24]. The ratio of males to females was close to 1:1 (292:283).

The information of these studies is summarized in Table 2. All of the patients were treated with oral sirolimus for at least 6 months. The initial dose given to most of the patients was 1 mg/m^2^/d once a day. One study [32] gave a starting dose of 0.5 mg/m^2^/d once a day, and another study [22] gave a starting dose of 0.8 mg/m^2^/d twice a day. In another study [25], the dose was calculated based on the patient weight, and this dose was 0.08 mg/kg/d twice a day. In addition, there were seven different targeted blood concentration ranges, which could be summarized into two subgroups. Five studies [25,26,31,32,35] were under 10 ng/mL, and one study [22] was over 10 ng/mL. The remaining three studies maintained targeted blood concentrations of 5–15 [31], 4–12 [25], or 4–13 ng/mL [32].

### 3.2. The Risk of Bias

The risk of bias was judged based on the NOS (Table 3). All of the studies scored nine, indicating good quality. For several AEs, such as oral mucositis and upper respiratory tract infection, the data from Iris EO et al. [28], Liern M et al. [19], and Ji Y et al. [17] were the main sources of bias, leading to an I2 larger than 90%. Removing these data reduced the heterogeneity and led to an I2 less than or close to 50%. For other AEs, the same method was adopted to reduce the heterogeneity and bias as much as possible.

### 3.3. Summary of the Incidence of AEs

All of the data regarding the incidence of AEs that occurred in one or more of the patients are listed in Table 4. Some major categories of AEs were divided into smaller categories on account of the difference in the frequencies in each study. If there was a cross, the maximum value was taken instead of adding them together. The incidence of all the AEs was less than 21%. Among them, oral mucositis had the highest incidence (20.52%) while cellulitis, muscle pain, dizziness, polyuria, and red eye had the lowest incidence (0.17%). Other AEs with an incidence ≥5% included upper respiratory tract infection (16.35%), gastrointestinal reaction (9.22%), increases in liver enzymes (9.22%), dyslipidemia (6.26%), pain (6.09%), and nausea and vomiting (5.22%).

Then, we statistically analyzed the AEs reported in more than two studies to make the incidence rates more robust. The incidence data before and after the removal of the highly heterogeneous biased data are detailed in Table 5. After the statistical analysis, oral mucositis was also the most common adverse event (21.9%, 95% CI: 0.112–0.325), and the incidence rate of upper respiratory tract infections was also greater than 20% (21.2%, 95% CI: 0.076–0.347). Moreover, the incidence rates of gastrointestinal reactions (14.5%, 95% CI: 0.044–0.245) and liver function damage (7.3%, 95% CI: 0.022–0.125) were more than 5%, which was the same as those before statistical analysis. However, the incidence rates of nausea and vomiting (4.8%, 95% CI: 0.000–0.110), dyslipidemia (4.7%, 95% CI: 0.027–0.067), and pain (3.3%, 95% CI: 0.003–0.064) decreased, compared with those before the statistical analysis, which was less than 5%. In the two subcategories of dyslipidemia, i.e., hyperlipidemia (3.9%, 95% CI: 0.021 to 0.057) and hypercholesterolemia (1.4%, 95% CI: 0.000 to 0.031), hyperlipidemia was more common. This may be due to the fact that hyperlipidemia included the increase in triglycerides and cholesterol (the statistical description after the removal of highly heterogeneous data is shown in Table S2).

### 3.4. Subgroup Analysis of AEs

It could be seen from the nine articles that we were able to conduct a subgroup analysis using two factors: the drug concentration in the blood and the diseases.

With regard to the targeted blood concentrations, 10 ng/mL was a boundary value. Five studies [25,26,31,32,35] treated patients with a targeted blood concentration of <10 ng/mL, whereas one study [22] used a value of ≥10 ng/mL. Through a statistical analysis of the AEs that at least one study in both groups had reported, the AEs with considerable differences (≥10%) between the two subgroups were oral mucositis (29.2%), gastrointestinal reaction (28.3%), pneumonia (16.7%), increases in liver enzymes (13.7%), and eczema (11.1%) (Table S3). After a further analysis, it was easy to observe that the results with statistical significance (*p* < 0.01) were gastrointestinal reaction, nausea and vomiting, diarrhea, oral mucositis, upper respiratory tract infection, pneumonia, eczema, neutropenia, and increases in liver enzymes. These findings suggest that these AEs were more likely to be seen in the high concentration group (≥10 ng/mL).

With regard to diseases, only one study reported nephrotic syndrome [24]. Therefore, we could divide the candidate studies into two categories. Four studies reported tuberous sclerosis complex [25,31,32,35] and four reported vascular anomalies [24,27,33,34]. After analysis, we found that oral mucositis was more likely to occur in patients with vascular anomalies (*p* < 0.01). The incidence of oral mucositis in the tuberous sclerosis group was 7.8% (95% CI: 0.049 to 0.107) while the incidence in the vascular anomaly group was 33.9.0% (95% CI: 0.207 to 0.472) (Table S4). Moreover, the incidences of several AEs in the two subgroups differed in value, although there were no significant differences. For instance, gastrointestinal reaction and acne were more common in the tuberous sclerosis complex group. In contrast, pneumonia and pain were more likely to occur in the vascular anomaly group.

### 3.5. Summary of the Severity of AEs

**Figure 2:**
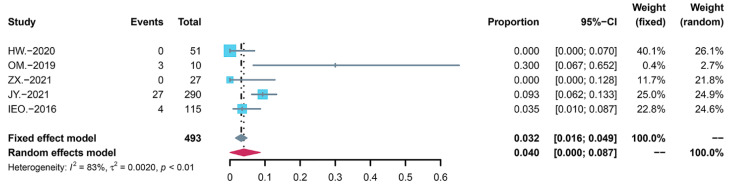
Forest map of serious adverse events before removal of the biased data.

**Figure 3:**
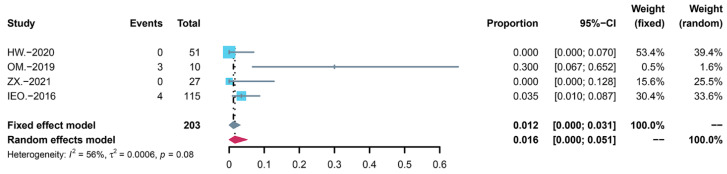
Forest map of serious adverse events after removal of the biased data.

According to the Common Terminology Criteria for Adverse Events (CTCAE), sirolimus toxicities are divided into five grades. The occurrence of AE severity discussed by studies is shown in Table 6. There were no deaths (Grade Ⅴ) related to AEs, but several patients had Grade Ⅳ AEs. Among the nine studies, four children in the study by Ji Y et al. [22] had grade IV AEs, including three pneumonitis and one upper respiratory infection. Two studies [31,33] reported grade III AEs, including four pneumonitis and one upper respiratory infection. We classified grade III and above as serious adverse events and performed statistical analysis. The incidence was 4.0% (95% CI: 0.000 to 0.087) before adjusting for heterogeneity (Figure 2), whereas the incidence changed to 1.6% (95% CI: 0.000 to 0.051) after the removal of the highly heterogeneous data (Figure 3). Therefore, even if the number of AEs was significant, most of them were mild, and no fatal AEs occurred.

### 3.6. Subgroup Analysis of the Severity

**Figure 4:**
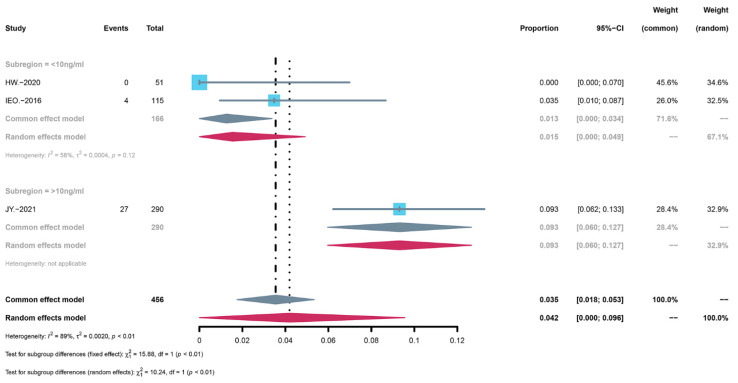
Forest map of the concentration subgroup analysis of serious adverse events.

**Figure 5:**
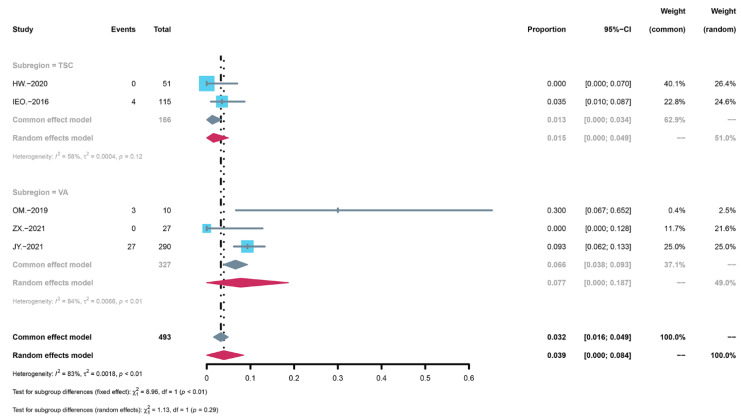
Forest map of the disease subgroup analysis of serious adverse events.

The results of the two subgroup analyses were different. In the concentration subgroup analysis, the result was statistically significant (*p* < 0.01) (Figure 4). Serious adverse reactions were more common in the high concentration group, and the incidence was 9.3% (95% CI: 0.060 to 0.127) while the incidence in the low concentration group was 1.5% (95% CI: 0.000 to 0.049). In contrast, there was no significant difference after the disease subgroup analysis (*p* = 0.29) (Figure 5). The incidence of vascular anomalies was 7.7% (95% CI: 0.000 to 0.187) while the incidence of tuberous sclerosis complex was 1.5% (95% CI: 0.000 to 0.049).

## 4. Discussion

In this study, for the first time, we systematically evaluated the AEs of sirolimus in children and indicated that sirolimus was safe under regular monitoring. Previously, limited data were available on the safety of sirolimus therapy in the pediatric population. Sandbank S et al. [8] retrospectively analyzed 150 children or young adults diagnosed with complicated vascular anomalies. The investigators found that sirolimus was effective in 85% of cases and could be well tolerated. Sirolimus appeared to be an effective and safe treatment. However, this was a descriptive analysis, and this study lacked objective statistical analysis.

In the present study, a detailed statistical analysis was conducted on prospective studies. In total, 575 children with different kinds of diseases were treated with oral sirolimus. Among them, 32 kinds of AEs occurred. The incidences of individual AEs were less than 21%. After the removal of the highly heterogeneous data, the incidence of individual AEs was less than 10%. The main adverse events were oral mucositis, upper respiratory tract infection, increases in liver enzymes, and dyslipidemia. These findings are similar to the studies by Nadal M et al. [20] and Sandbank S et al. [8]. In addition, the incidence of several AEs was related to the sirolimus targeted blood concentrations, such as gastrointestinal reaction, nausea and vomiting, diarrhea, oral mucositis, upper respiratory tract infection, pneumonia, eczema, neutropenia, and increases in liver enzymes. In other words, AEs were more likely to appear when the concentration was maintained at ≥10 ng/mL than at <10 ng/mL. This indicated that the AEs associated with sirolimus might be dose dependent. On the premise of ensuring a curative effect, low-dose sirolimus should be used to reduce the potential side effects; for example, low-dose sirolimus treatment should be used for kaposiform hemangioendothelioma [37]. In patients who develop discrete proteinuria and/or canker sores during sirolimus treatment, the symptoms usually disappeared after reducing the dose of sirolimus. Although some scholars have proposed this view, there has been no prospective study to determine the dose of sirolimus in children. In this case, 0.8 mg/m2/d was a low dose. However, in order to determine the method of administering the drug and what dose is needed, more research is required.

More interestingly, we also found that the type of disease could affect the occurrence of some AEs. Oral mucositis was more frequently reported in children with vascular anomalies than in those with tuberous sclerosis complex. This may be associated with the different effects of drugs on diseases. Oral mucositis probably results from the direct toxic effects of sirolimus on mucosal membranes and might be dose dependent. However, researchers currently pay more attention to the toxic effects of sirolimus on islets or hepatocytes [38,39], and there is no detailed study on oral mucositis, which should be our future research direction.

With regard to the severity of the AEs, the disease could not magnify the severity of AEs, but increases in the targeted blood concentration could increase the incidence of serious AEs. Most of the AEs were mild and nonlethal. However, in some cases, the severity of AEs reached Grades III and IV. The most common serious AE was pneumonitis.

Sirolimus-induced pneumonitis was first described in renal patients in 2000 [40]. Unlike infectious pneumonia with pathogens, drug-induced pneumonia is an inflammatory reaction of the lung that is caused by drugs and their metabolites through direct cytotoxicity and allergic reactions. Most of its pathogenesis is unclear. Avitzur Y et al. [41] reported the first case of interstitial granulomatous pneumonitis associated with sirolimus in pediatric orthotopic liver transplantation. This patient had no respiratory symptoms and only imaging evidence, and the targeted blood concentration was relatively low (4–6 ng/mL). This finding suggested that patients with relatively low sirolimus blood concentrations also need monitoring. It was also stressed that repeated pulmonary function tests measured before and during sirolimus treatment might be an effective means to monitor the development of sirolimus-induced pneumonitis [41]. Another study by Frexio C et al. [18] reported that two fatal pulmonary infections occurred in two patients with vascular anomalies (with ages of 1 month and 6 months) receiving sirolimus treatment. This review also evaluated antibiotic prophylaxis with trimethoprim-sulfamethoxazole, which has been advocated by some scholars. The incidence of infection decreased from 5.2% to 2.5% compared with patients without prophylaxis [18]. Consequently, during sirolimus treatment, continuous and regular pulmonary function tests are needed to evaluate the respiratory status of pediatric patients to prevent the occurrence of fatal pneumonitis. The preventive use of sulfamethoxazole may be an effective method, but no RCTs or registered clinical trials were found in this regard. Thus, further research is needed to confirm the benefit of sulfamethoxazole.

Why is pneumonitis the most serious adverse reaction to sirolimus? Perhaps this is because the symptoms of pneumonitis can develop acutely or insidiously [42]. In the early stage, the toxicity of sirolimus could expose cryptic antigens produced by the autoimmune response [40], and this is often asymptomatic and subclinical. When symptoms are detected, serious clinical manifestations emerge, and there is acute development of pneumonitis. Although multiple mechanisms of sirolimus-induced lung injury have been explored, the pathogenesis is not clearly known. Therefore, we can only reduce the incidence of pneumonitis through the abovementioned regular pulmonary function tests.

There were several limitations in our study. First, sirolimus is approved for patients over 13 years old. Although many studies have reported sirolimus treatment in children, only two RCTs were available. The other studies were single-arm studies. This led to the systematic analysis of single-arm research. Second, there was only one study in the high concentration group, so the results of this study determined the results of the final statistical analysis. This may have caused serious bias. Therefore, more studies are needed to verify whether higher targeted blood concentrations will increase the incidence of AEs. Third, although the highly heterogeneous data were removed, the I^2^ was still high, thus indicating a high degree of heterogeneity. This might lead to deviations in the results.

## 5. Conclusions

Oral sirolimus in the treatment of childhood diseases is safe and reliable. Although most of the AEs were mild and nonlethal, some AEs could still reach Grade III or above. Therefore, we recommend that sirolimus treatment should be strictly monitored to reduce the occurrence of serious or fatal AEs. The AEs associated with sirolimus may be dose dependent. On the basis of ensuring curative effects, a low dose of sirolimus should be adopted. Whether our findings have clinical significance needs further study. More prospective studies, especially RCTs, are needed to assess the safety of sirolimus in children.

## Figures and Tables

**Table 1:** List of studies identified and selected through database searches: demographic characteristics.

First Author	Year of Publication	Country	The Source of Funding	Study Type	Patients (*n*)	Age		Sex, *n*		Diseases
						Median (Year)	Range (Year)	Male	Female	
Cardamone, M [23]	2014	Australia	Novartis	Single-center open-label	7	6	3–17	3	4	Tuberous sclerosis complex
Chen, X. Q. [29]	2021	China	The National Key Research and Development Program of China (No. 2016YFC1000707) and The National Natural Science Foundation of China (No. 81471329)	Prospective cohort study	217	6	2–23	121	96	Tuberous sclerosis complex
He, W. [30]	2020	China	The National Key Research and Development Program of China (2016YFC1000707)	Prospective cohort study	91	2	0–12	47	44	Tuberous sclerosis complex
Ozeki, M. [31]	2019	Japan	A Clinical research-clinical trial promotion research project (18lk0201055h0003) and Practical Research Project for Rare/Intractable Diseases (18ek0109277h0002)	Prospective study	12	6.5	0.04–18	6	6	Lymphatic anomalies
Zhang, X. [32]	2021	China	Beijing Hospitals Authority’ Ascent Plan (DFL20191201) and Beijing Hospitals Authority Youth Program (QML20181202)	Prospective open-label study	27	2.3	0–15	12	15	Lymphatic anomalies
Liern, M. [24]	2012	Argentina	-	Prospective cohort study	13	10	8–18	4	9	Nephrotic syndrome
Ji, Y. [22]	2021	China	The National Natural Science Foundation of China (81400862 and 81401606), the Key Project in the Science & Technology Program of Sichuan Province (2019YFS0322), etc.	Multicenterphase II trial	126	4.8	0–14	64	62	Vascular anomalies
Iris E. Overwater [33]	2016	Netherlands	The Dutch Epilepsy Foundation	Randomized controlled study	23	5.5	1.8–10.9	11	12	Tuberous sclerosis complex
Marua, A. [25]	2021	France	The French Ministry of Social Affairs and Health (French National Program of Clinical Research [PHRC-N], 2014)	Randomized controlled study	59	11.6y	6–18y	24	35	Slow-flow vascular malformations
Summary					575			292	283	

**Table 2:** List of studies identified and selected through database searches: intervention.

First Author	Intervention			Treatment Duration	
	Starting Dose	Regimen	Targeted Blood Concentration	Range	Median
Cardamone, M [23]	1 mg/m^2^ /d	—	4–10 ng/ml	6–36 months	18 months
Chen, X. Q. [29]	1 mg/m^2^ /d	—	5–10 ng/ml	7–22 months	13 months
He, W. [30]	1 mg/m^2^ /d	qd	5–10 ng/ml	—	—
Ozeki, M. [31]	BSA ≥ 1.0 m^2^ 2 mg/d BSA< 1.0 m^2^ 1 mg/d	qd	5–15 ng/ml	6–30 months	12.5 months
Zhang, X. [32]	0.5 mg/m^2^ /d	qd	4–13 ng/ml	6–27 months	10.6 months
Liern, M. [24]	1 mg/m^2^ /d	qd	7–10 ng/ml	12 months	12 months
Ji, Y. [22]	0.8 mg/m^2^	bid	10–15 ng/ml	0.4–4.5 years	3 years
Iris E. Overwater [33]	—	—	5–10 ng/ml	6 months	6 months
Marua, A. [25]	0.08 mg/kg/d	bid	4–12 ng/ml	12 months	12 months

**Table 3:** List of the quality of all studies based on the NOS ^a^.

Studies	Selection				Comparability		Outcome			Total Quality Score	Level ^b^
Author	Exposed Cohort	Nonexposed Cohort	Ascertainment of Exposure	Outcome of Interest Not Present at Star	Main Factor	Additional Factor	Assessment of Outcome	Follow-Up Long Enough	Adequacy of Follow-Up of Cohorts		
Cardamone, M [23]	1	1	1	1	1	1	1	1	1	9	Good
Chen, X. Q. [29]	1	1	1	1	1	1	1	1	1	9	Good
He, W. [30]	1	1	1	1	1	1	1	1	1	9	Good
Ozeki, M. [31]	1	1	1	1	1	1	1	1	1	9	Good
Zhang, X. [32]	1	1	1	1	1	1	1	1	1	9	Good
Liern, M. [24]	1	1	1	1	1	1	1	1	1	9	Good
Ji, Y. [22]	1	1	1	1	1	1	1	1	1	9	Good
Iris E. Overwater [33]	1	1	1	1	1	1	1	1	1	9	Good
Marua, A. [25]	1	1	1	1	1	1	1	1	1	9	Good

^a^ NOS: Newcastle Ottawa Scale. ^b^ Poor (score, 0–3), fair (score, 4–6), or good (score, 7∼9).

**Table 4:** Summary of adverse events.

	Sirolimus (Total Patients *n* = 575)	
	*n* ^a^	%
Patients with at least 1 adverse event		
Oral mucositis	118	20.52
Acne	25	4.35
Pneumonia	26	4.52
Upper respiratory tract infection	94	16.35
Lymph node infection	5	0.87
Otitis media	2	0.35
Other infection	12	2.09
Fever	6	1.04
Gastrointestinal reaction	53	9.22
♦ Nausea and vomiting	30	5.22
♦ Diarrhea	13	2.26
Anorexia	6	1.04
Cellulitis	1	0.17
Rash	10	1.74
Eczema	17	2.96
Pain	35	6.09
♦ Headache	17	2.96
♦ Muscle pain	1	0.17
Dizziness	1	0.17
Hypertension	4	0.70
Edema	4	0.70
Hemorrhagic disease	4	0.70
Fatigue	4	0.70
Alopecia	5	0.87
Hyperhidrosis	3	0.52
Polyuria	1	0.17
Wound healing delay	4	0.70
Red eye	1	0.17
Behavioral change	3	0.52
Injury due to accident	4	0.70
Laboratory		
Dyslipidemia	36	6.26
♦ Hypercholesterolemia	17	2.96
♦ Hyperlipidemia	23	4.00
♦ Elevated LDL	7	1.22
Anemia	6	1.04
Neutropenia	15	2.61
Lymphocytopenia	8	1.39
Thrombocytosis	25	4.35
Increases in liver enzymes	53	9.22
♦ spartate aminotransferase raised	3	0.52
♦ Alanine aminotransferase raised	2	0.35

^a^ If there is a cross, the maximum value shall be taken (specifically, choosing the minimum number of patients with possible adverse events).

**Table 5:** List of the incidence rates of adverse events before and after the removal of highly heterogeneous data.

Adverse Events ^a^	Incidence Rate			
	Before Deletion		After Deletion ^b^	
	I^2^	Incidence Rate ^c^	I^2^	Incidence Rate ^c^
Gastrointestinal reaction	95%	14.5% (95%CI: 0.044–0.245)	25%	0.1% (95%CI: 0.000–0.007)
♦ Nausea and vomiting	89%	4.8% (95%CI: 0.000–0.110)	0%	0.1% (95%CI: 0.000–0.007)
♦ Diarrhea	71%	1.4% (95%CI: 0.000–0.036)	0%	0.0% (95%CI: 0.000–0.006)
Oral mucositis	91%	21.9% (95%CI: 0.112–0.325)	32%	8.2% (95%CI: 0.054–0.110)
Acne	91%	3.8% (95%CI: 0.000–0.076)	26%	0.1% (95%CI: 0.000–0.007)
Upper respiratory tract infection	96%	21.2% (95%CI: 0.076–0.347)	51%	3.5% (95%CI: 0.000–0.082)
Pneumonia	78%	2.2% (95%CI: 0.000–0.050)	0%	0.0% (95%CI: 0.000–0.006)
Anorexia	0%	0.1% (95%CI: 0.000–0.006)	0%	0.1% (95%CI: 0.000–0.006)
Fatigue	0%	0.0% (95%CI: 0.000–0.005)	0%	0.0% (95%CI: 0.000–0.005)
Pain	84%	3.3% (95%CI: 0.003–0.064)	71%	1.5% (95%CI: 0.000–0.038)
♦ Headache	65%	0.6% (95%CI: 0.000–0.021)	0%	0.0% (95%CI: 0.000–0.005)
Edema	0%	0.1% (95%CI: 0.000–0.007)	0%	0.1% (95%CI: 0.000–0.007)
Alopecia	0%	0.1% (95%CI: 0.000–0.007)	0%	0.1% (95%CI: 0.000–0.007)
Eczema	62%	1.4% (95%CI: 0.000–0.035)	0%	0.0% (95%CI: 0.000–0.006)
Dyslipidemia	0%	4.7% (95%CI: 0.027–0.067)	0%	4.7% (95%CI: 0.027–0.067)
♦ Hypercholesterolemia	61%	1.4% (95%CI: 0.000–0.031)	50%	0.1% (95%CI: 0.000–0.007)
♦ Hyperlipidemia	0%	3.9% (95%CI: 0.021–0.057)	0%	3.9% (95%CI: 0.021–0.057)
Anemia	0%	0.2% (95%CI: 0.000–0.008)	0%	0.2% (95%CI: 0.000–0.008)
Neutropenia	54%	1.9% (95%CI: 0.000–0.045)	0%	0.1% (95%CI: 0.000–0.007)
Increases in liver enzymes	71%	7.3% (95%CI: 0.022–0.125)	35%	5.3% (95%CI: 0.029–0.078)

^a^ ≥2 articles reported the adverse event. ^b^ Deletion of highly heterogeneous articles until the I^2^ ≤ 50% or as close as possible to 50% was achieved. ^c^ If I^2^ > 50%, we used random effects. If I^2^ ≤ 50%, we chose fixed effects.

**Table 6:** List of grades of all adverse events.

First Author	Patients (*n*)	Grades (*n*)		
		All	Grade Ⅰ–Ⅱ	Grade Ⅲ–Ⅳ
He, W.	91	51	51	0
Ozeki, M.	12	10	7	3
Zhang, X.	27	27	27	0
Ji, Y.	126	290	263	27
Iris E. Overwater	23	115	111	4

## Supplementary Materials

The following supporting information can be downloaded at: https://www.mdpi.com/article/10.3390/children9091295/s1, Table S1: a list of studies removed after reading the main text; Table S2: description of the incidence of AEs after removing highly heterogeneous data; Table S3: list of incidence rates of adverse events after targeted blood concentration subgroups analysis; Table S4: list of incidence rates of adverse events after disease subgroups analysis; Table S5: PRISMA_2020_checklist; Table S6: PRISMA_2020_flow_diagram.
