# Supplementary material for: Safety Evaluation of Oral Sirolimus in the Treatment of Childhood Diseases: A Systematic Review
Source: Children (Basel). 2022 Aug 26;9(9):1295. doi: 10.3390/children9091295 (PMC9497617; doi:10.3390/children9091295)
Supplement: Supplementary file 1 [file children-09-01295-s001.zip › Supplementary Table 1 A list of studies removed after reading the main text.pdf]

A list of studies removed after reading the main text

Reason	n
Age≥18 or identified as a adult study	15
Topical therapy	7
Retrospective study	9
Case report	6
Drug eluting stent therapy	2
Not clear data	3
Animal therapy	2
Mainly for clearance rate	2
Not in English	2
Total	48

A list of studies removed after conforming to the inclusion criteria and exclusion criteria

Title	Author	Reason
Histological progression of chronic renal allograft injury comparing sirolimus and mycophenolate mofetil-based protocols	Blydt-Hansen T.D.	Only present a table of the number of times rather than the number of cases
Sirolimus for progressive neurofibromatosis type 1-associated plexiform neurofibromas: a neurofibromatosis Clinical Trials Consortium phase II study	Brian Weiss, B.C.	Only show the adverse events data of the experimental and placebo groups together
Safety and pharmacokinetics of ascending single doses of sirolimus (Rapamune, rapamycin) in pediatric patients with stable chronic renal failure undergoing dialysis	Tejani A.	Mainly discuss patient tolerance to four sirolimus doses
