# Supplementary material for: Safety Evaluation of Oral Sirolimus in the Treatment of Childhood Diseases: A Systematic Review
Source: Children (Basel). 2022 Aug 26;9(9):1295. doi: 10.3390/children9091295 (PMC9497617; doi:10.3390/children9091295)
Supplement: Supplementary file 1 [file children-09-01295-s001.zip › Supplementary Table 2 Description of the incidence of AEs after removing highly heterogeneous data.pdf]

Description of the incidence of AEs after removing highly heterogeneous data

All of the incidence rates decreased after removing the highly heterogeneous data. Oral mucositis was also the most common adverse event (8.2%, 95% CI: 0.054 to 0.110). Other common AEs included liver function damage (5.3%, 95% CI: 0.029 to 0.078), dyslipidemia (4.7%, 95% CI: 0.027 to 0.067) and upper respiratory tract infection (3.5%, 95% CI: 0.000 to 0.082). In the two subcategories of dyslipidemia, i.e., hyperlipidemia (3.9%, 95% CI: 0.021 to 0.057) and hypercholesterolemia (0.2%, 95% CI: 0.000 to 0.007), hyperlipidemia was the most common, the same as the result before removing highly heterogeneous data. Even though many kinds of AEs occurred, the incidence rates were less than 10% after the statistical analysis.
