# Supplementary material for: Safety Evaluation of Oral Sirolimus in the Treatment of Childhood Diseases: A Systematic Review
Source: Children (Basel). 2022 Aug 26;9(9):1295. doi: 10.3390/children9091295 (PMC9497617; doi:10.3390/children9091295)
Supplement: Supplementary file 1 [file children-09-01295-s001.zip › Supplementary Table 3 List of incidence rates of adverse events after targeted blood concentration subgroups analysis.pdf]

Supplementary Table S3 List of incidence rates of adverse events after targeted blood concentration subgroups analysis

Adverse events [a]	Targeted blood drug concentration (Incidence rate)		
	<10ng/ml	≥10ng/ml	P-value
	Incidence rate [b]	Incidence rate [b]	
Gastrointestinal reaction	1.9% (95%CI: 0.000-0.055)	30.2% (95%CI: 0.221-0.382)	<0.01
♦ Nausea and vomiting	0.1% (95%CI: 0.000-0.007)	6.0% (95%CI: 0.000-0.159)	<0.01
♦ Diarrhea	0.0% (95%CI: 0.000-0.007)	8.7% (95%CI: 0.038-0.137)	<0.01
Oral mucositis	8.1% (95%CI: 0.052-0.109)	37.3% (95%CI: 0.289-0.457)	<0.01
Acne	14.1% (95%CI: 0.000-0.421)	6.3% (95%CI: 0.021-0.106)	0.59
Upper respiratory tract infection	18.9% (95%CI: 0.000-0.379)	26.2% (95%CI: 0.185-0.339)	<0.01
Pneumonia	0.0% (95%CI: 0.000-0.006)	16.7% (95%CI: 0.102-0.232)	<0.01
Anorexia	1.7% (95%CI: 0.000-0.048)	0.0% (95%CI: 0.000-0.011)	0.31
Edema	0.0% (95%CI: 0.000-0.006)	1.6% (95%CI: 0.000-0.038)	0.17
Alopecia	0.0% (95%CI: 0.000-0.006)	3.2% (95%CI: 0.001-0.062)	0.05
Eczema	0.0% (95%CI: 0.000-0.006)	11.1% (95%CI: 0.056-0.166)	<0.01
Dyslipidemia	3.9% (95%CI: 0.013-0.065)	6.3% (95%CI: 0.021-0.106)	0.34
♦ Hypercholesterolemia	3.7% (95%CI: 0.000-0.090)	0.0% (95%CI: 0.000-0.011)	0.19
♦ Hyperlipidemia	0.1% (95%CI: 0.000-0.007)	6.3% (95%CI: 0.021-0.106)	0.07
Anemia	0.1% (95%CI: 0.000-0.007)	1.6% (95%CI: 0.000-0.038)	0.20
Neutropenia	0.1% (95%CI: 0.000-0.007)	8.7% (95%CI: 0.038-0.137)	<0.01
Increases in liver enzymes	6.1% (95%CI: 0.030-0.092)	19.8% (95%CI: 0.129-0.268)	<0.01
serious adverse events	1.5% (95%CI: 0.000-0.049)	9.3% (95%CI: 0.060-0.127)	<0.01

[a] Each subgroup ≥1 articles reported the adverse event.
[b] If I^2 >50%, we used random effects. If I^2≤50%, we chose fixed effects.
