# Supplementary material for: Safety Evaluation of Oral Sirolimus in the Treatment of Childhood Diseases: A Systematic Review
Source: Children (Basel). 2022 Aug 26;9(9):1295. doi: 10.3390/children9091295 (PMC9497617; doi:10.3390/children9091295)
Supplement: Supplementary file 1 [file children-09-01295-s001.zip › Supplementary Table 4 List of incidence rates of adverse events after disease subgroups analysis.pdf]

Supplementary Table S4 List of incidence rates of adverse events after disease subgroups analysis

Adverse events [a]	Diseases (Incidence rate)		
	Vascular anomalies	Tuberous sclerosis complex	P-value
	Incidence rate [b]	Incidence rate [b]	
Gastrointestinal reaction	1.9% (95%CI: 0.000-0.079)	24.8% (95%CI: 0.00-0.631)	0.25
♦ Nausea and vomiting	10.8% (95%CI: 0.000-0.318)	1.1% (95%CI: 0.000-0.041)	0.37
Oral mucositis	33.9% (95%CI: 0.207-0.472)	7.8% (95%CI: 0.049-0.107)	<0.01
Acne	2.6% (95%CI: 0.000-0.073)	17.9% (95%CI: 0.000-0.533)	0.40
Upper respiratory tract infection	24.1% (95%CI: 0.117-0.365)	24.3% (95%CI: 0.000-0.650)	0.99
Pneumonia	9.2% (95%CI: 0.000-0.209)	0.0% (95%CI: 0.000-0.007)	0.12
Anorexia	0.0% (95%CI: 0.000-0.011)	2.1% (95%CI: 0.000-0.057)	0.28
Edema	1.2% (95%CI: 0.000-0.033)	0.0% (95%CI: 0.000-0.006)	0.29
Alopecia	2.0% (95%CI: 0.000-0.046)	0.0% (95%CI: 0.000-0.006)	0.14
Eczema	3.9% (95%CI: 0.000-0.114)	0.0% (95%CI: 0.000-0.007)	0.31
Fatigue	0.0% (95%CI: 0.000-0.011)	0.0% (95%CI: 0.000-0.007)	0.99
Pain	9.8% (95%CI: 0,000-0.221)	0.0% (95%CI: 0.000-0.006)	0.12
♦ Headache	6.0% (95%CI: 0.000-0.183)	0.0% (95%CI: 0.000-0.006)	0.34
Dyslipidemia	5.0% (95%CI: 0.025-0.075)	4.3% (95%CI: 0.016-0.069)	0.69
♦ Hypercholesterolemia	2.0% (95%CI: 0.000-0.056)	5.0% (95%CI: 0.000-0.121)	0.46
♦ Hyperlipidemia	3.7% (95%CI: 0.000-0.088)	4.2% (95%CI: 0.021-0.064)	0.85
Anemia	1.2% (95%CI: 0.000-0.033)	0.1% (95%CI: 0.000-0.007)	0.31
Neutropenia	3.3% (95%CI: 0.000-0.093)	0.1% (95%CI: 0.000-0.007)	0.49
Increases in liver enzymes	10.5% (95%CI: 0.000-0.213)	6.8% (95%CI: 0.036-0.100)	0.52
serious adverse events	7.7 (95%CI: 0.000-0.187)	1.5% (95%CI: 0.000-0.049)	0.29

[a] Each subgroup ≥1 articles reported the adverse event.

[b] If I^2 >50%, we used random effects. If I^2≤50%, we chose fixed effects.
